# Regulation of the JNK3 Signaling Pathway during Islet Isolation: JNK3 and *c-fos* as New Markers of Islet Quality for Transplantation

**DOI:** 10.1371/journal.pone.0099796

**Published:** 2014-07-01

**Authors:** Saida Abdelli, Klearchos K. Papas, Kate R. Mueller, Mike P. Murtaugh, Bernhard J. Hering, Christophe Bonny

**Affiliations:** 1 Departement of Medical Genetics, Centre Hospitalier Universitaire Vaudois (CHUV) and University of Lausanne, Lausanne, Switzerland; 2 Department of Surgery, University of Arizona, Institute for Cellular Transplantation, Tucson, Arizona, United States of America; 3 Department of Veterinary and Biomedical Sciences, St. Paul, University of Minnesota, Minneapolis, Minnesota, United States of America; 4 Department of Surgery, Schulze Diabetes Institute, University of Minnesota, Minneapolis, Minnesota, United States of America; University of Lille Nord de France, France

## Abstract

Stress conditions generated throughout pancreatic islet processing initiate the activation of pro-inflammatory pathways and beta-cell destruction. Our goal is to identify relevant and preferably beta-specific markers to assess the activation of beta-cell stress and apoptotic mechanisms, and therefore the general quality of the islet preparation prior to transplantation. Protein expression and activation were analyzed by Western blotting and kinase assays. ATP measurements were performed by a luminescence-based assay. Oxygen consumption rate (OCR) was measured based on standard protocols using fiber optic sensors. Total RNA was used for gene expression analyzes. Our results indicate that pancreas digestion initiates a potent stress response in the islets by activating two stress kinases, c-Jun N-terminal Kinase (JNK) and p38. JNK1 protein levels remained unchanged between different islet preparations and following culture. In contrast, levels of JNK3 increased after islet culture, but varied markedly, with a subset of preparations bearing low JNK3 expression. The observed changes in JNK3 protein content strongly correlated with OCR measurements as determined by the *Spearman's* rank correlation coefficient *rho* 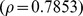 in the matching islet samples, while inversely correlating with *c-fos* mRNA expression 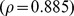. In conclusion, pancreas digestion recruits JNK and p38 kinases that are known to participate to beta-cell apoptosis. Concomitantly, the islet isolation alters JNK3 and *c-fos* expression, both strongly correlating with OCR. Thus, a comparative analysis of JNK3 and *c-fos* expression before and after culture may provide for novel markers to assess islet quality prior to transplantation. JNK3 has the advantage over all other proposed markers to be islet-specific, and thus to provide for a marker independent of non-beta cell contamination.

## Introduction

Human islet transplantation (HIT) may substitute for insulin therapy promising a better control of blood glucose levels, and therefore, potentially limiting inherent secondary complications in type 1 diabetic patients [1]–[4]. This alternative method is currently limited to only a restricted number of patients as it currently requires more than two pancreas donors per recipient, and long-term immunosuppressive treatment [3]–[5]. The process of the islet isolation from pancreas donors usually fails to produce sufficient functional islet mass (FIM) for transplantation [3], [6], [7]. Indeed, pancreas procurement, enzymatic digestion and mechanical purification damage the tissue and reduce beta-cell function and viability, compromising long-term islet graft function [8]–[10]. Furthermore, a significant number of implanted islets are lost immediately following transplantation, while those surviving the initial non-specific insults are exposed to alloimmune and autoimmune attacks by the immune system.

HIT suffers from a lack of reliable markers and easy-to-perform tests for predicting potential loss of graft function or islet mass in a timely manner [11]–[13]. Several methods have been proposed to test the qualitative and quantitative characteristics of islet preparations [13]–[16]. Few of the currently performed tests can consistently predict outcomes following islet transplantation [14], [17]. Recently, it has been shown that an assay based on islet oxygen consumption rate (OCR) may replace the *in vivo* Nude Mouse Bioassay (NMB) [18]–[21]. The NMB consists of monitoring diabetes reversal after infusion of islets into the kidney capsule of nude mice that received a diabetogenic dose of streptozotocin for 30 days prior to establish an outcome [11], [13], [21]. The OCR method which can be completed within 1–2 hours is operator independent and has recently been shown to be able to predict clinical islet auto- and allotransplantation outcomes with high specificity and sensitivity [18]–[20]. However, identification of pro-inflammatory and stress markers in islet preparations at the molecular level may provide additional critical information about the quality of the islet products.

Islet loss occurs mainly by apoptotic mechanisms recruiting different death-signaling pathways including the Mitogen-Activated Protein Kinases (MAPKs) c-Jun N-terminal Kinases (JNK) and p38 [10], [22]–[24]. Three JNK protein kinases have been described: JNK1 and JNK2 are ubiquitously expressed, while JNK3 is restricted to the brain and pancreatic islets [25], [26]. To date, no islet-specific isoform has been reported for p38 [27]. JNKs are activated by the upstream kinases MAPK-Kinase 4 (MKK4) and MKK7 and regulate the phosphorylation of various substrates including the nuclear transcription factors c-Jun and c-Fos [28]–[30]. These factors belong to the immediate early genes (IEGs) as their expression is induced immediately following MAPK activation [30], [31].

We recently determined that in contrast to JNK1 and JNK2, JNK3 has an anti-apoptotic activity in insulin-secreting cells [26], [32]. JNK3 acts through preserving the activation of the insulin receptor substrate 2 (IRS2) and the Akt/protein kinase B (PKB) signaling pathway [32]. Three Akt isoforms (PKBα/Akt1, PKBβ/Akt2, and PKBγ/Akt3) have been identified [33]–[35]. Activation of Akts is regulated by the upstream signaling IRS-phosphoinositide 3-kinase (PI3-K) that requires both protein phosphorylation and membrane translocation. Activated Akts phosphorylate an array of substrates including the Glycogen Synthase Kinase 3β (GSK3β) among many others and mediate several important downstream effects like cell proliferation, survival, mitosis, and protein synthesis [24], [35]–[37].

No studies have focused on the level of expression of the beta-specific isoform JNK3 as a marker of islet stress and damage following the isolation process. JNK3 protein has been shown to positively influence the pro-survival network of insulin-secreting cells [26], [32]. The present study investigates the potential use of JNK3 and other molecular markers to assess islet quality prior to transplantation. We demonstrate that both JNK3 and *c-fos* can provide for reliable indicators of the general stress levels generated throughout the isolation procedure.

## Materials and Methods

### Ethics Statement

All animal experiments were approved by the Institutional Animal Care and Use Committee (IACUC) at the University of Minnesota (Protocol approval #0501A66650) and conducted according to the National Institutes of Health guidelines.

### Islet preparation and culture

Adult male landrace porcine pancreases were procured and islets were isolated at the University of Minnesota isolation laboratory. Samples were collected from a series of eighteen *(P_1–18_)* pig pancreases during and following the islet isolation procedure using a modified Ricordi method [38]. The islet equivalents (IE) and purity (>90%) were determined by dithizone staining. Purified (*D_0_*) islets were kept in culture for seven days *(D_7_)* in ME199 media (Mediatech) supplemented with 10% [vol./vol.] heat-inactivated porcine serum (HIPS, Gibco, Auckland, New Zealand), L-glutamine (Mediatech) and heparin (10 U/ml, APP Pharmaceuticals) at 37°C in humidified air without CO_2_.

### JNK kinase assays and densitometric quantification

Islet pellets were dislodged into cold lysis buffer from Cell Signaling Technologies (CST, USA) supplemented with protease inhibitor cocktail tablets (Roche Applied Science, Basel, Switzerland). The homogenized tissue was sonicated and whole protein extracts were recovered by cold centrifugation (12000× *g* for 30 min). Protein extracts (50 µg) were incubated for 3 hours at 4°C with a c-Jun (1–79)-GST fusion protein coupled to glutathione beads (1 µg, Sigma Aldrich, Switzerland). After a short centrifugation (900× *g* for 3 min), the supernatants were discarded, and pellets were washed twice, then mixed to kinase buffer (20 mmol/l HEPES pH 7.5, 20 mmol/l β-glycero-phosphate, 10 mmol/l MgCl_2_, and 1 mmol/l DTT) and incubated (30 min at 30°C) with 0.5 µl ^[γ-33]^ATP (111 TBq/mmol, PerkinElmer, Switzerland). The reactions were terminated by addition of Laemmli sample buffer (50 mmol/l Tris-HCl pH 6.8, 2% [w./vol.] SDS, 100 mmol/l DTT, 0.1% [w./vol.] bromophenol blue and 10% [vol./vol.] glycerol. GST-c-Jun phosphorylation was resolved by SDS-PAGE, (BIO-RAD, Switzerland) and then, gels were fixed, dried and exposed overnight to X-ray film. The protein band intensities were quantified by densitometric analysis using the ImageJ processing software.

### Western-blotting

Whole protein extracts (25–30 µg) without preliminary immunoprecipitation were resolved by SDS-PAGE and gels were electroblotted onto nitrocellulose membranes. The blots were probed overnight with the following primary antibodies against: phospho (p)-p38, p38, p-ERKs, ERKs, JNK3, JNK1, p-Akt1, p-Akt2, Akts, p-GSK3β, MKK7, PTEN, p-c-Jun and c-Jun (1∶1000, CST, USA). Equal protein loading was ascertained by blotting membranes with antibodies against glyceraaldehyde-3-phosphate dehydrogenase (GAPDH, 1∶3000, CST, USA) or tubulin (1∶5000, Sigma Aldrich, Switzerland). Anti-rabbit and anti-mouse infrared (IR)-labeled secondary antibodies (1∶10000, CST, USA) were used to detect protein bands and fluorescence intensities were quantified with IR-Imaging Software (LI-COR Odyssey, Biosciences GMBH, Germany).

### RNA extraction and quantification of c-fos mRNA expression by real-time RT-PCR

RNA was extracted using the RNeasy minikit (Qiagen) following the manufacturer's instructions. 2 µg of total RNA was converted to cDNA with the Quantitect Reverse Transcription kit (Qiagen). We applied perfecta SYBR Green FastMix for qRT-PCR (Quanta Biosciences, Foster City, CA) using specific primer sequences [39]. Quantitative RT-PCR was performed on an ABI-7500 machine with the following parameters; 95°C for 1 minute, then 50 cycles at 95°C for 4 seconds and 60°C for 45 seconds, followed by a dissociation step. Each sample was run in triplicate. The internal control housekeeping gene was cyclophilin. Negative control was RNAse-free water. Data were recorded as the mean *Ct* value normalized to the cyclophilin mean.

### OCR measurements

Islet samples were assessed for OCR in triplicate (∼1000 IE each) in water-jacketed, air tight titanium chambers (Instech Laboratories, Plymouth Meeting, PA, USA) equipped with optic fiber sensors that measure the oxygen partial pressure (pO_2_) over a time as described previously [18], [19], [40]. The OCR measurements (change in nmol O_2_ per unit of time) were conducted in culture media equilibrated at 37°C.

### ATP measurements

The Cell Titer-Glo Luminescent Cell Viability Assay (Promega Corp, Madison, Wisc) was used per manufacturer's instructions to measure ATP. Islet sample preparations for the analysis were performed as described previously [14]. Triplicate of islet-cell suspensions (100 µl) were taken from each islet preparation and diluted 10-fold in Dulbecco's phosphate buffered saline (DPBS, Mediatech, Herndon, Va). The islet cells were sonicated at amplitude of 11% for 15 seconds (Fisher Scientific, Sonic Dismemberator Model 500). Samples prepared according the instructions provided by the kit and they were plated in 96-white-well plates (Corning 3912, Corning Inc.) for luminescence readings on a Spectra Max M5 plate reader (Molecular Devices). Serially diluted ATP was used as a standard (Sigma-Aldrich).

### DNA content measurements

Islet samples that were analyzed for ATP and OCR were diluted 10-fold in an aqueous solution containing 1 mol/l ammonium hydroxide (Mallenckrodt, St. Louis, Mo) and 3.4 mmol/l Triton X-100. The DNA content was then determined using the Quant-iT PicoGreen dsDNA assay kit per manufacturer's instructions. Briefly, islet suspensions were sonicated at amplitude of 11% for 15 seconds (Fisher Scientific, Sonic Dismemberator Model 500) and stained with a fluorescent DNA stain. Fluorescence was read with a 96-well fluorometer (excitation at 480 nm, fluorescence emission intensity measured at 520 nm) and compared to a standard curve (Quant-iT PicoGreen dsDNA kit, Molecular Probes, Eugene, OR). OCR and ATP were normalized by the DNA content to determine OCR/DNA (nmol O_2_/min-mg•DNA) and ATP/DNA (nmol/mg•DNA) ratios.

### Statistical analysis

We analyzed pancreas and islet specimens from eighteen *(P_1–18_)* killed adult pigs. In each case, the purity of islet preparations was between 90% and 95%. All experiments were performed a minimum of five times (n = 5–18) in duplicate or triplicate. For some experiments, the eighteen specimens were examined (n = 18) results are presented as means ± SEM. The two-tailed *Student's t* test was used to determine the statistical significance when compared two groups. For multiple comparisons, one-way analysis of variance (ANOVA) was performed. The differences found between the experimental groups were considered statistically significant if ^*^P<0.05, ^**^P<0.01 and ^***^P<0.001.

The correlation study was determined by the *Spearman's* rank correlation coefficient *rho*. The analysis has been performed to examine if there is an association between the two variables OCR and JNK3 (n = 18 and 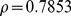); test of Ho: delta *(d)-OCR* and *d-JNK3* are independent *(Prob>|t| = 0.0001)* or between OCR and *c-fos* (n = 6 and 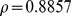); test of Ho: *d-OCR* and *d-fos* are independent *(Prob>|t| = 0.0188)*. We used the *Spearman's* correlation method as the relationship between variables is not linear and the analyzed variables are not normally distributed.

## Results

### MAPKs levels: Activation of JNKs and p38s following pancreas enzymatic digestion

**Figure 1:**
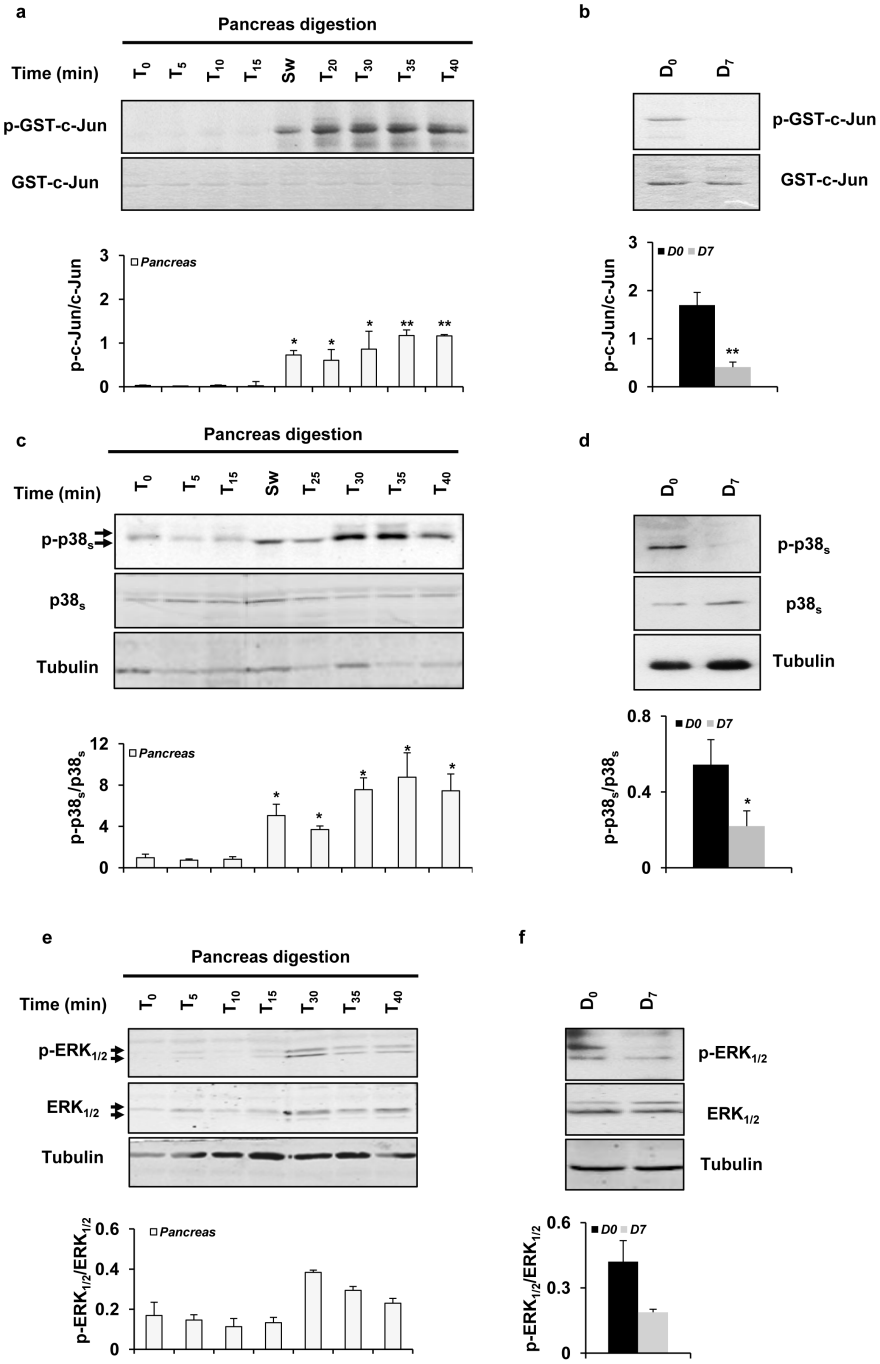
Effect of the islet isolation procedure on activation of MAPKs. *(* ***a/b*** *)* For measuring JNK activation by JNK assays, protein extracts (50 µg) were mixed with the GST-Jun fusion protein (GST-c-Jun) and used as described in *“* *Materials and Methods* *”* Photographs represent ^γ33^P-phosphorylation of the substrate (p-GST-c-Jun) following SDS-PAGE. The levels of *(* ***c/d*** *)* p38 and *(* ***e/f*** *)* ERK_1/2_ protein phosphorylation and expression were determined by Western blot using antibodies against: phospho (p)-p38, p38, p-ERK_1/2_ and ERK_1/2_. The band density values were calculated as a ratio of *(* ***a/b*** *)* p-GST-c-Jun normalized to GST-c-Jun, *(* ***c/d*** *)* p-p38s normalized to p38s and *(* ***e/f*** *)* p-ERK_1/2_ normalized to ERK_1/2_. Results are means ± SEM of five to thirteen separate experiments (n = 5–13) and are presented as graphics: white bars; pancreas (>98% exocrine tissue), black bars; purified *D_0_* islets (90% endocrine tissue) and grey bars; cultured *D_7_* islets (>90% endocrine tissue). *(* ***a/b*** *)* JNK activation: Statistically significant differences are assessed by ANOVA or Student's *t*-test with ^*^P<0.05 or ^**^P<0.01 for all groups prior *vs.* after SW (n = 5) and for *D_0_ vs. D_7_* islets (n = 13). No significant differences are found among all groups from *T_0_* to *T_15_*. *(* ***c/d*** *)* p38 activation: Differences are significant for groups *T_0_–_15_* compared to *T_sw_-_40_* during pancreas digestion (n = 5, ^*^P<0.05) and for *D_0_ vs. D_7_* islets (n = 13, ^*^P<0.05). *(* ***e/f*** *)* No significant differences are found among all groups.

We first assessed the activation levels of the three MAPKs JNKs, p38s and ERKs during the process of islet isolation. Samples were collected at different times (Time points *T_0–40_*_ min_) throughout the digestion procedure. Islets were further collected at the end of the isolation (*D_0_*) and after seven days (*D_7_*) of culture. Samples were used for protein preparation to perform JNK assays and Western blot analyzes (Fig. 1). Activation of both stress kinases JNK (Fig. 1a) and p38 (Fig. 1c) was almost undetectable from the time points *T_0–15_*_ min_ but progressively increased after the switch point (*SW*), persisting up to the end of the pancreatic digestion process (>98% exocrine tissue). At the end of the isolation process, JNK (Fig. 1b) and p38 (Fig. 1d) activation was high in *D_0_* islets, and then declined to low baseline levels in *D_7_* islets (n = 13, ^**^P<0.01 for JNK and ^*^P<0.05 for p38). Similarly, activation of ERK_1/2_ slightly increased from *T_30 min_* and remained stable throughout the digestion procedure (Fig. 1e). At *D_0_*, ERK_1/2_ activation was slightly higher than what observed in *D_7_* islets (Fig. 1f). Collectively, the data indicate that the enzymatic digestion process initiates a marked and sustained activation of the two stress kinases JNK and p38. This has profound implication for islet-cell function and viability.

### The islet isolation procedure alters JNK3 protein content

**Figure 2:**
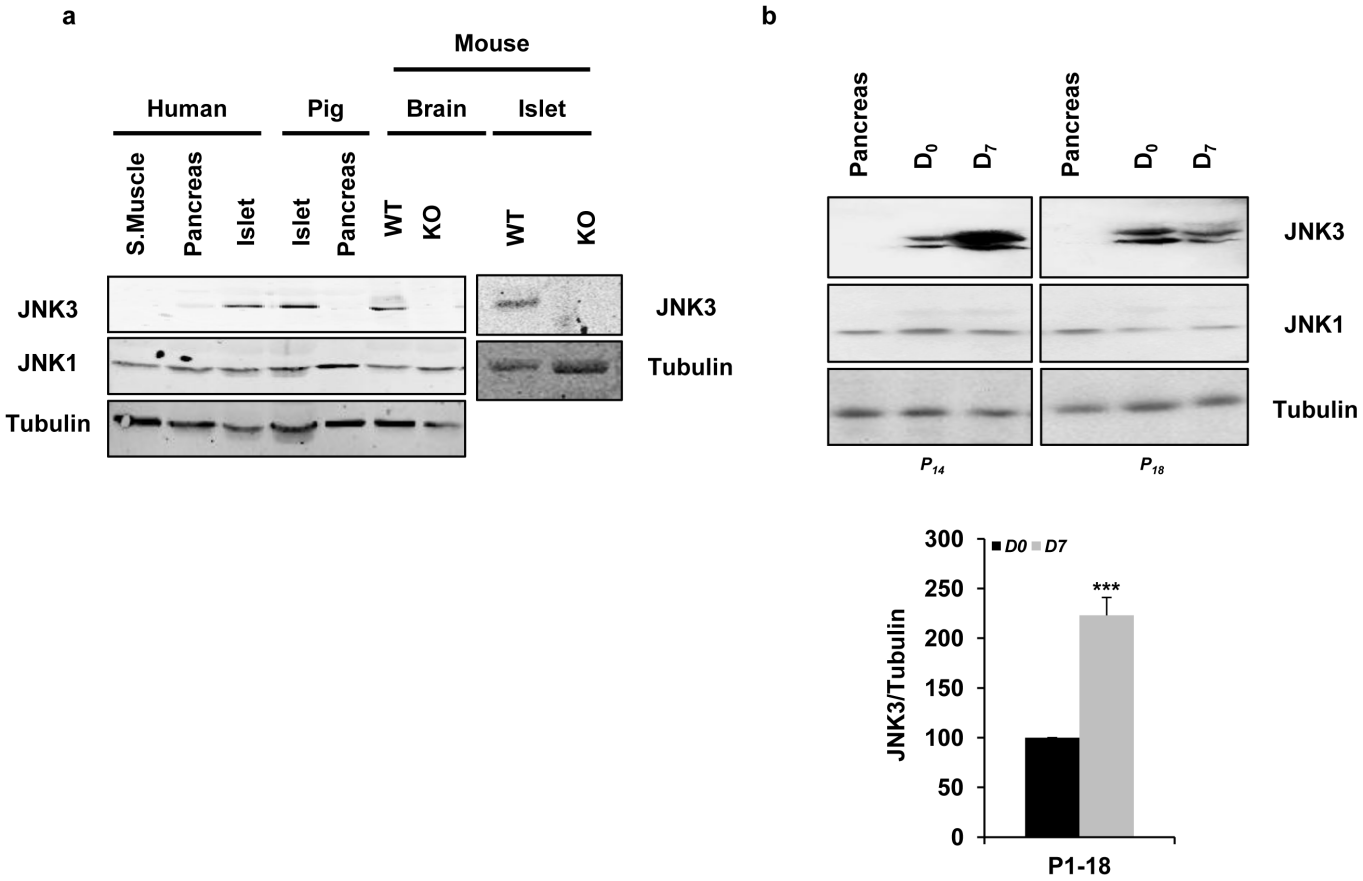
JNK3 expression analysis. *(* ***a/b*** *)* Protein extracts (30 µg) were prepared from tissue of different species (human, pig and mouse): skeletal muscle (S. Muscle), pancreas, brain and islets generated from wild type (*WT*) and *Jnk3*-knockout (*KO*) mice. JNK3 expression levels were examined by Western blot in *D_0_* and *D_7_* using a JNK3 monoclonal antibody. JNK1 and tubulin monoclonal antibodies were used as loading controls. Protein bands were scanned and quantified by Odyssey scan software. The values were summarized in graphics. Data are means ± SEM of eighteen independent experiments (n = 18, ^***^P<0.001 for JNK3 in *D_0_* (black bars) *vs. D_7_* (grey bars)).

JNK3 is mostly expressed in the brain and, as we recently demonstrated [26], is also highly expressed in human, pig and mouse pancreatic islets (Fig. 2a). JNK3 protein levels were low in purified islets at *D_0_* but increased after *D_7_* culture in most preparations tested (n = 18, ^***^P<0.001) (Fig. 2b). However, in a subset of islet preparations *(P_2_, P_5_, P_7_, P_11_, P_13_, and P_18_)*, JNK3 remained similarly low in both conditions. In marked contrast, JNK1 protein expression levels remain unaffected, independently of the islet preparation (Fig. 2b). Collectively, the data show that the stress of the isolation procedure alters specifically JNK3 protein content in purified islets.

### *In vitro* culture allows islet recovering and enhances islet metabolic function

**Figure 3:**
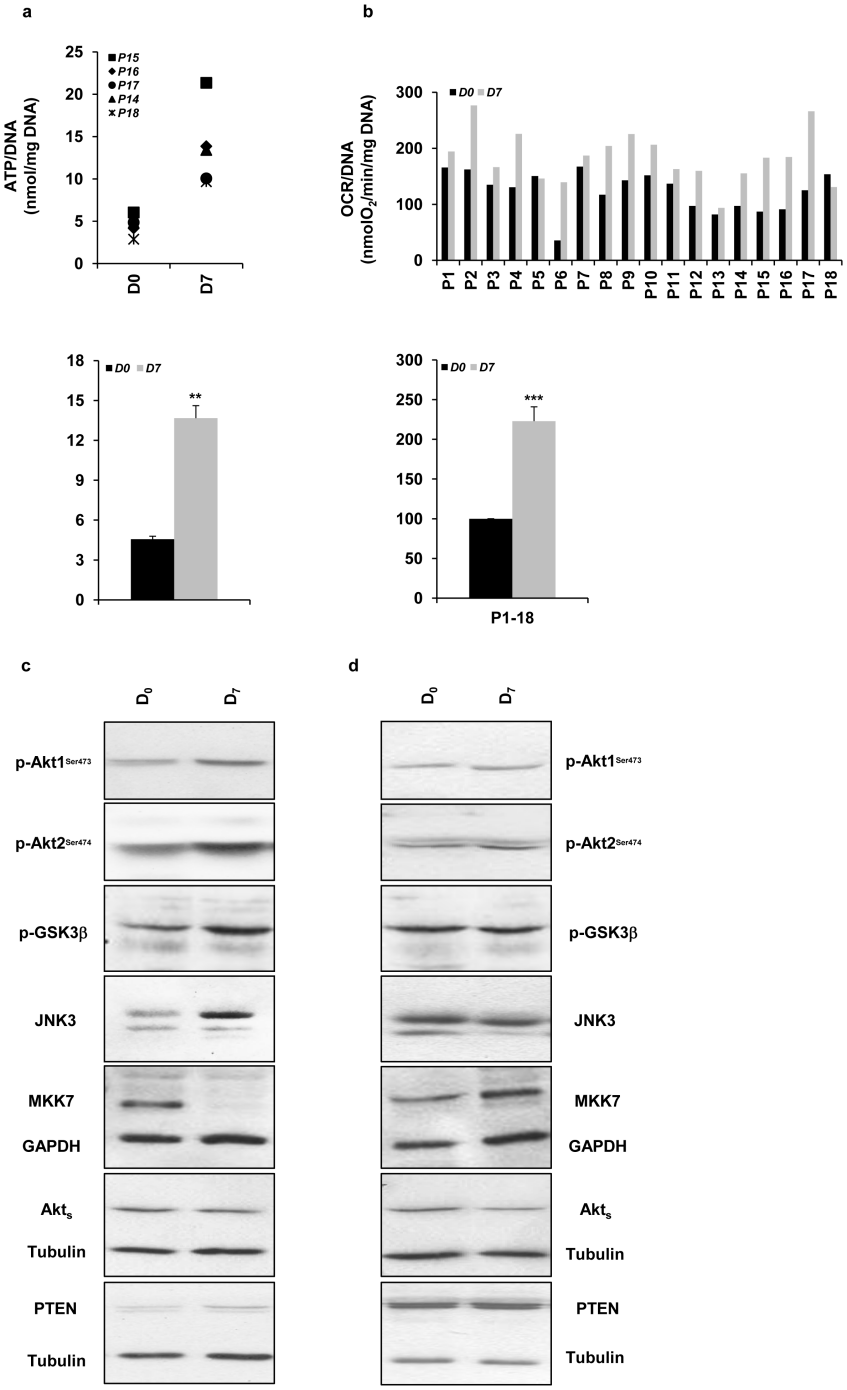
Islet culture improves islet viability by increasing ATP content and OCR. *(* ***a*** *)* ATP was measured and normalized to DNA content (nmol/mg protein/mg-DNA) as described in *“* *Materials and Methods* *”*. Graphic represents ATP/DNA values. Results are means ± SEM of five separate experiments performed in triplicate (n = 5, ^**^P<0.01 for *D_0_* (black bars) *vs. D_7_* (grey bars)). *(* ***b*** *)* Islet samples were used for OCR measurements normalized to DNA content (nmolO_2_/min/mg-DNA) as described in *“* *Materials and Methods* *”*. Graphic represents OCR/DNA values. Data are means ± SEM of eighteen separate experiments performed in triplicate (n = 18, ^***^P<0.001 for *D_0_ vs. D_7_*). *(* ***c/d*** *)* Protein extracts (30 µg) from islet samples with *(* ***c*** *)* high or *(* ***d*** *)* low JNK3 protein content in *D_0_* and *D_7_* were tested by Western blot. Polyclonal antibodies against: p-Akt1, p-Akt2, p-GSK3β, JNK3, MKK7, Akts and PTEN were used. Equal protein loading was assessed by blotting membranes with antibodies against tubulin and GAPDH. Data are representative of at least three separate experiments (n = 3–8).

Culturing isolated islets for a few days may allow for the damaged beta-cells to recover. Accordingly, *in vitro* culture drastically enhanced islet-cell function by increasing the levels of ATP/DNA in all islet preparations tested (n = 5, ^**^P<0.01) (Fig. 3a). Interestingly, we observed that the islet preparation that showed the lowest value of ATP/DNA (*P_18_*, Fig. 3a) did not restore JNK3 protein content at *D_7_* (Fig. 2b). Similarly, OCR/DNA measured in both *D_0_* and *D_7_* islets frequently increased in *D_7_* islets (n = 18, ^***^P<0.001) except in some preparations where it decreased (*P_18_*), or remained low (*P_5_ and P_13_*) (Fig. 3b); in these conditions, JNK3 protein content mostly stayed unaffected or decreased (Fig. 2b). Furthermore, we frequently observed that culturing islets enhanced the activation of Akt1 and Akt2 and their downstream substrate GSK3β, concomitantly to increasing JNK3 protein levels (Fig. 3c). Interestingly, the JNK upstream kinase MKK7 became undetectable in *D_7_* islets (Fig. 3c), whereas its expression was found in the majority of islet preparations that showed low or unchanged JNK3 expression levels (Fig. 3d). Frequently, in those preparations, Akt1 and Akt2 activities did not increase following islet culture (Fig. 3d), while their activities were significantly enhanced (increased p-GSK3β) when JNK3 expression increased in *D_7_* islets. Akts and PTEN protein expression levels were still unchanged in both conditions as determined by the levels of control loading, GAPDH and tubulin. These data further describe the beneficial effect of culturing isolated islets by enhancing the pro-survival JNK3-Akt1/2-GSK3β signaling pathways as well as down-regulating the pro-apoptotic factors MKK7-JNK1/2.

### Downstream of JNKs:Islet isolation modulates nuclear c-Jun levels

**Figure 4:**
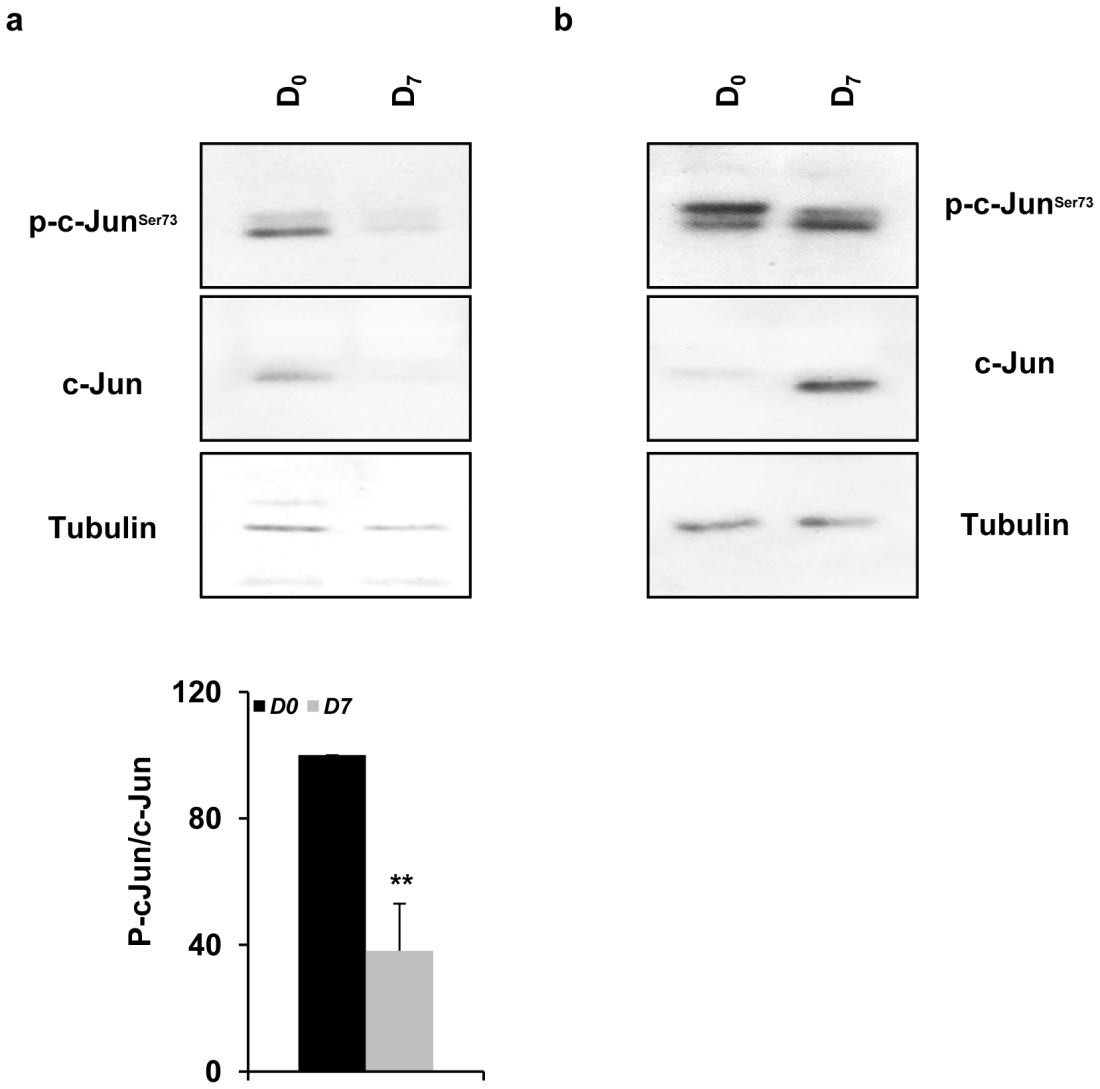
Islet isolation alters c-Jun levels. *(* ***a/b*** *)* Protein extracts (25–30 µg) from islet samples *D_0_* and *D_7_* were analyzed by Western blot using the following antibodies against c-Jun and p-c-Jun. Samples with *(* ***a*** *)* low (basal state) and *(* ***b*** *)* high (induced) p-c-Jun/c-Jun protein levels in *D_0_* and *D_7_* were analyzed. Equal protein loading was assessed by blotting membrane with an anti-tubulin antibody. Protein bands were scanned and quantified by Odyssey scan software. The values were presented as a single graphic. All values are means ± SEM of thirteen separate experiments (n = 13, ^**^P<0.01 for *D_0_ vs. D_7_*).

Sustained expression of IEGs is mainly related to the severity of organ damage and graft failure in organ transplantation [41]. Therefore, we examined by Western blot the levels of c-Jun activation and expression in thirteen (*P_1–13_*) islet preparations. As expected, c-Jun activation (required for initiating its transcriptional regulation) and expression were increased in all islet samples tested at *D_0_* (n = 13, ^**^P<0.01) and frequently reduced to very low or undetectable levels at *D_7_* (Fig. 4a). Unexpectedly, in four islet preparations, *(P_13_, P_16_, P_17_ and P_18_)*, phosphorylated c-Jun and c-Jun stayed high at *D_7_* (Fig. 4b) despite decreased JNK activation levels in the matching islet preparations (n = 13, ^**^P<0.01). The fraction (50%) of islets that retained high c-Jun/phospho-c-Jun levels did not restore high JNK3 protein content at *D_7_ (P_13_ and P_18_)*. This observation suggests that c-Jun is not a reliable marker for islet quality assessment.

### Correlation between increased OCR and increased JNK3 protein content and decreased c-fos mRNA expression following islet culture

**Figure 5:**
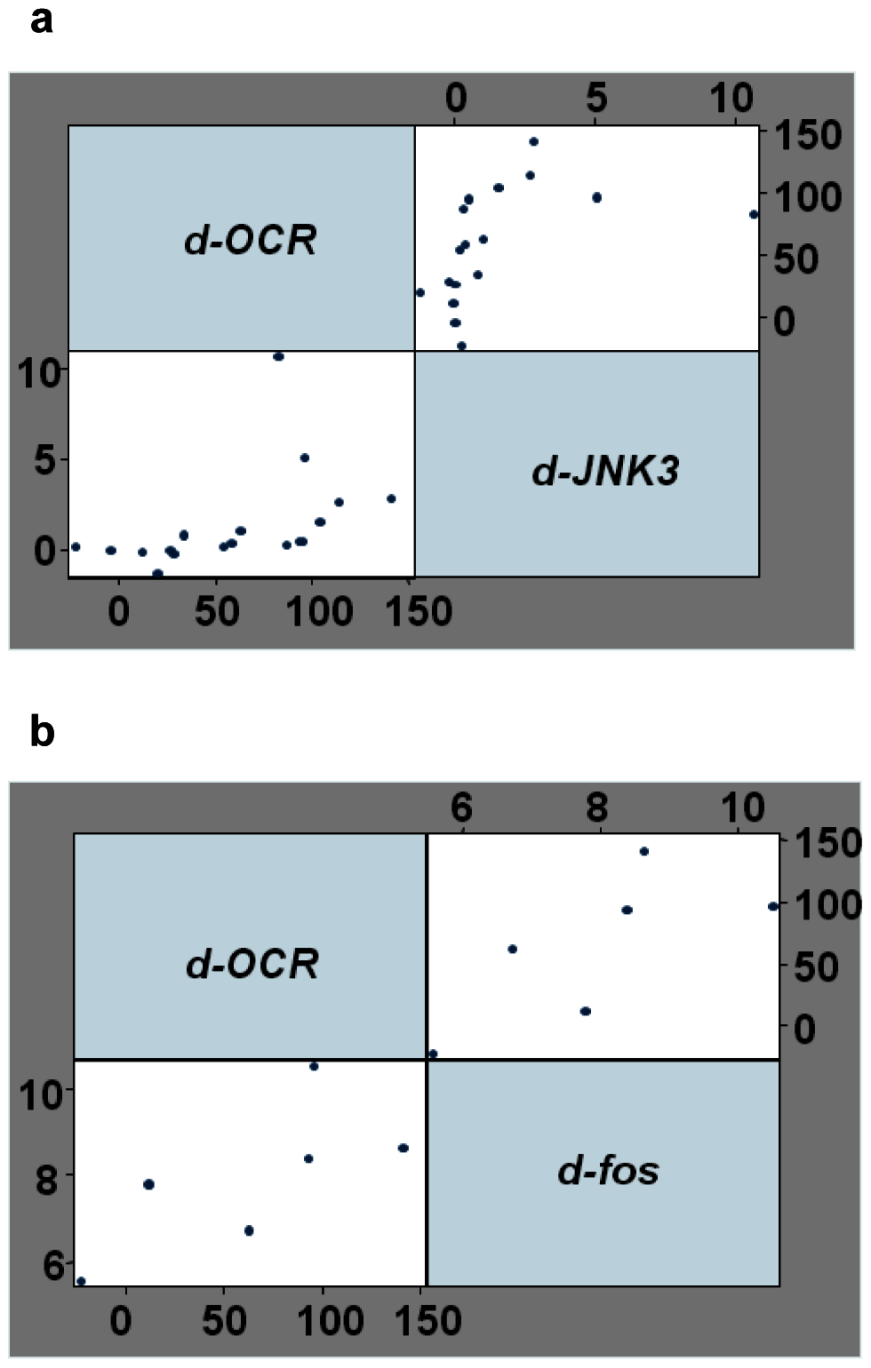
JNK3 and c-fos strongly correlate to OCR. Correlation analysis using *the Spearman's* correlation coefficient *rho* was determined as described in *“Statistical analysis”* by plotting the values of delta *(d)*-OCR_D7-D0_ together with *(* ***a*** *) d-JNK3* (n = 18, ^***^P<0.001, 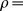
0.7853) and with *(* ***b*** *) d-fos* (n = 6, 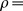
0.8857).

We aimed to establish whether JNK3 expression levels could be used to assess islet quality. We measured both JNK3/Tubulin and OCR/DNA ratios in eighteen *(P_1–18_)* islet preparations and determined whether there was a correlation between the values of OCR at *D_0_ vs. D_7_* and the values of JNK3 in similar conditions. The analysis was completed by calculating the difference *D_7_-D_0_ (delta)-OCR (d-OCR)* and *D_7_-D_0_ d-JNK3* in *(P_1–18_)* islet preparations and variables were analyzed using the *Spearman's* correlation method. The data determined that the increase in *d-OCR* closely correlated with the increase of *d-JNK3* (n = 18, ^***^P<0.001 and 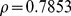) (Fig. 5a). Similarly, we established an inverse correlation between increasing *d-OCR* and decreasing *d-fos* mRNA expression (n = 6 and 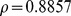) (Fig. 5b). Collectively, these data indicate that JNK3 and *c-fos* correlated well to OCR and thus, can be used as reliable markers to assess islet quality prior to transplantation.

## Discussion

Preserving islets prior to and after transplantation is a complex issue including hypoxia generated during pancreas procurement, inflammation and mechanical damage induced by the isolation process, the effects of exocrine cell products released and instant blood-mediated inflammatory reaction (IBMIR) following islet transplantation [7], [9], [42], [43]. The intensity of these events collectively impaired FIM in human recipients; consequently, more than two pancreas donors per recipient are needed to achieve insulin-independence [3], [5].

Here, we show that both stress-activated protein kinases JNK and p38 are recruited in the early hours following the initiation of the pancreas digestion. The magnitude of their activation is associated with low viability at the end of the islet isolation procedure (decreased ATP and OCR). It has been reported as well that both isolation and extended period of culture progressively depleted islets from their heparin sulfate stores that correlated with increased beta-cell death [44]. Accordingly, heparin was added to the culture medium to improve islet resistance and minimize islet aggregation; this heparin treatment also reduces islet loss due to the effects of IBMIR following transplantation [45]. Previous studies have shown that culturing islets decreased the expression of stress and apoptotic markers and suppressed immunogenicity [39]. Furthermore, we determined that culturing islets increased ATP/DNA and consequently, enhanced the insulin secretion capacity; it also brought the benefit of down-regulating the activity of the recruited stress-kinases (mainly the pro-apoptotic MKK7-p-JNK1/2-p-c-Jun/*c-fos* and p-p38s) to the basal levels and accordingly, allowing islets to recover before their grafting. Meanwhile, culturing islets enhanced the activity of the pro-survival Akts-GSK3β signaling pathway. Importantly, we establish that both JNK3 (but not JNK1) and *c-fos* levels are altered at *D_0_ vs. D_7_*; these changes tightly correlated with OCR/DNA [18], [19]. Remarkably, OCR measurements at *D_7_* are higher than at *D_0_*, resulting in improved viability of islets. This is critical because the OCR/DNA value is predictive of graft outcome.

Early pre-transplant assays, applied to assess islet function and viability, are required to predict graft failure [13], [14], [40]. The current study describes additional “molecular” markers, JNK3 and *c-fos* as novel markers predictive of islet quality. Both markers tightly correlated with OCR/DNA (but not with ATP/DNA), an assay that has been proposed to be of general use for measuring islet potency prior to transplantation [13], [18]. In this study, we have shown the advantages of testing JNK3/*c-fos* levels in addition to other functional assays (ATP or OCR). Measuring JNK3/*c-fos* can be fast, easily performed and routinely applied in clinical trials with no need of specific equipment to run the tests (Western-blotting-based/quantitative PCR). Most importantly, JNK3 is specific to beta-cells and when compared to other viability tests, its represents a selective marker in the pancreatic preparations that discriminates beta-cell mass from the non-beta-cell tissue fraction. In fact, islet preparations are heterogeneous, with contaminating fractions of exogenous and non-beta-immune cells that complicate the testing. Most potency tests used for islet characterization are poorly reproducible and usually overestimate the total amount of viable islets, and thus, fail to predict the graft outcome [13], [40], [46]. Indeed, combination of several assays which are materials- (requiring high number of islets) and time-consuming is required to validate islet preparations for transplantation [13]. Currently, the best available assay which correlates well with clinical outcomes is the NMB, which cannot be used as a predictive tool due to the long follow-up of the mice required (over 30 days). Presently, OCR measurements are certainly one of the most promising assays that correlate with transplant outcomes in the NMB [18], [40], [46]. However, OCR is not specific to beta-cells and therefore is critically dependent upon purity of the preparations.

It has been shown that the induction of the IEGs such as *c-jun* or *c-fos* is strongly associated with the initiation of apoptosis in many cells and tissues as well as in pancreatic beta-cells [23], [47], [48]. In particular, continuous expression of IEGs has been associated with graft failure [23], [41]. The islet isolation procedure induces stresses that are concordant with the observed changes in the molecular profiles of IEGs, (*i.e.* increased *c-fos* and *c-jun* levels). In all islet preparations analyzed in this study, JNK activation considerably decreased at *D_7_* (Fig. 1b), which consequently reduced c-Jun phosphorylation and expression in all but the four mentioned islet samples (*P_13_, P_16_, P_17_ and P_18_*, Fig. 4b). We presume that isolation stress involves the activation of myriad and complex intracellular signaling pathways; the sustained activation and expression of c-Jun may result from low turnover of c-Jun (slow degradation) and thus, increasing the protein stability through culture conditions.

The role of *c-fos* in apoptosis has been addressed in our previous studies using beta-cell lines. In particular, we showed that *c-fos* mRNA expression was also induced by the isolation process, and that culturing islets or blocking the JNK signaling pathway enhanced the viability of rat and human islets [23], [49], [50]. Here we have correlated *c-fos* mRNA expression (but not c-Jun) to OCR (strong correlation). Testing *c-fos* levels after the islet isolation and prior to transplant might further strengthen the results obtained with JNK3 to sense and quantify islet damage. Unexpectedly, c-Jun assessment was not correlated with OCR or ATP. It is well-known that c-Jun has opposite effects depending on stress conditions and or cell types [30]. Certainly, compared to JNK3 and *c-fos*, c-Jun seems to be unreliable marker for islet quality assessments.

### Conclusions

Assessment of the islet quality is required to improve successful islet transplantation. Therefore, identification of novel factors that efficiently predict the extent of islet damage is crucial for successful grafting. We propose that JNK3 and *c-fos* represent additional useful (together with OCR) markers to discriminate between healthy and poor islet quality preparations. JNK3 has the added benefit (over *c-fos* and OCR) of being an islet-specific marker. Further work will aim at translating these findings into a useful “kit” to help improving transplantation outcomes in human.
